# Letter to the editor: Increase of influenza vaccination coverage rates during the COVID-19 pandemic and implications for the upcoming influenza season in northern hemisphere countries and Australia

**DOI:** 10.2807/1560-7917.ES.2021.26.50.2101143

**Published:** 2021-12-16

**Authors:** Marco Del Riccio, Bruno Lina, Saverio Caini, Lisa Staadegaard, Sytske Wiegersma, Jan Kynčl, Béhazine Combadière, Chandini Raina MacIntyre, John Paget

**Affiliations:** 1 Netherlands Institute for Health Services Research (Nivel), Utrecht, Netherlands; 2 Postgraduate School in Hygiene and Preventive Medicine, University of Florence, Florence, Italy; 3 VirPath Laboratory, University of Lyon, Lyon, France; 4 Department of Infectious Diseases Epidemiology, National Institute of Public Health, Prague, Czech Republic; 5 Inserm, Centre d'Immunologie et des Maladies Infectieuses, Sorbonne Université, France; 6 Kirby Institute, University of New South Wales, Sydney, New South Wales, Australia

**Keywords:** Influenza vaccination, coverage rates, COVID-19, SARS-CoV-2, influenza

**To the editor:** We read with interest the collection of articles ‘Spotlight influenza 2021’ recently published in *Eurosurveillance* [1]. This collection highlights some important features of the unusual 2020/21 northern hemisphere influenza season and the accompanying editorial recommends preparedness measures for the upcoming 2021/22 season [2]. The authors suggest that vaccination may have contributed to the low influenza circulation in 2020/21 and use the example of Spain where influenza vaccination coverage rates (VCRs) increased across different target groups during the 2020/21 winter [2]. We would like to bring further evidence regarding this point by providing VCR data published by 10 northern hemisphere countries and Australia (Table), with a particular focus on elderly people.

**Table:** Influenza VCRs in the elderly by influenza season and difference between the first COVID-19 pandemic and the pre-pandemic season in 10 northern hemisphere countries and 1 southern hemisphere country, 2017/18 to 2020/21

**Northern hemisphere countries**							
**Country**	**Age (years)**	**2017/18 VCR (%)**	**2018/19 VCR (%)**	**2019/20 VCR (%)**	**2020/21 VCR (%)**	**2019/20–2020/21 difference (%)**	**Source**
England	≥65	73.0	72.0	72.4	80.9	+ 8.5	Public Health England
France		49.7	51.0	52.0	59.9	+ 7.9	Public Health France
Israel		59.8	59.2	59.8	68.2	+ 8.4	Ministry of Health, Israel; Organisation for Economic Co-operation and Development (OECD), France
Italy		52.7	53.1	54.6	65.3	+ 10.7	Ministry of Health, Italy
The Netherlands		60.4	60.3	61.3	67.9	+ 6.6	The Netherlands Institute for Health Services Research (Nivel)
Philippines^a^	≥60	NA	NA	2.6	5.6	+ 3.0	Ministry of Health, Philippines
Poland	≥65	13.0	14.2	15.1	18.4	+ 3.3	National Institute of Public Health, Poland
South Korea		81.3	82.8	83.8	73.6	− 10.2	Korea Disease Control and Prevention Agency, reported by Kwon Y et al. [5]
Spain		55.7	54.3	54.7	67.7	+ 13.0	Ministry of Health, Spain
United States		59.6	68.1	69.8	75.2	+ 5.4	Centers for Disease Control and Prevention (CDC)
**Southern hemisphere country**							
**Country**	**Age (years)**	**2017 VCR (%)**	**2018 VCR (%)**	**2019 VCR (%)**	**2020 VCR (%)**	**2019–2020 difference (%)**	**Source**
Australia	≥ 65	33.6	50.4	60.6	69.6	+ 9.0	Department of Health, Australia

VCR: vaccination coverage rate; NA: not available.

^a^ The Philippines is a tropical climate country with influenza activity occurring from June to November, and the vaccine is administrated from February on each year.

During the 2020/21 winter, influenza VCRs in elderly people increased in England (+ 8.5%), France (+ 7.9%), Israel (+ 8.4%), Italy (+ 10.7%), the Netherlands, (+ 6.6%), the Philippines (+ 3.0%), Poland (+ 3.3%), Spain (+ 13.0%), and the United States (US) (+ 5.4%), compared with 2019/20 (Table). Remarkably, England and the US managed to reach coverage rates of 75% in this population group, which is recommended by the World Health Organization (WHO) and the EU Council Recommendation 2009/1019/EU [3,4]. A decrease in VCRs was instead observed in South Korea (from 83.8% to 73.6%): this is likely related to a report of alleged influenza vaccine-related deaths that attracted considerable media attention in 2020 [5]. Of note, VCRs in most of these northern hemisphere countries remained generally stable in previous winters (Table). In the southern hemisphere, influenza VCRs in elderly people increased in Australia by 9.0% in 2020 compared with 2019. A further, slight increase was observed in 2021, while decreases were seen in other age groups [6]. These declines were possibly due to the convergence of influenza and coronavirus disease (COVID-19) vaccinations in 2021, with guidelines to space these apart by at least 2 weeks.

There has been very little influenza activity around the world since April 2020 [7] and it is difficult to anticipate influenza circulation in the upcoming winter. Current modelling scenarios range from a mild season to a large increase in cases, mainly depending on the extent of the use of non-pharmaceutical interventions (NPIs) [8]. The emergence of the acute respiratory syndrome coronavirus 2 (SARS-CoV-2) Omicron variant of concern means there will likely be a continuation of containment measures related to the COVID-19 pandemic – such as physical distancing, recommendations to wear face masks, and movement restrictions – which could result in late or little influenza virus activity in the northern hemisphere. We agree with Larrauri and Prosenc [2] that the recent low levels of influenza activity may lead to an increased proportion of susceptibles and predispose to severe epidemics at some point, so achieving high influenza VCRs will be crucial to contain the influenza burden, especially when NPIs are relaxed. Finally, strengthening influenza VCR monitoring in different population groups [9] will be critical to ensure that we are achieving – or at least approaching – the objectives we set for influenza prevention and control.

## References

[1] Eurosurveillance. Spotlight influenza 2021. [Accessed: 9 Dec 2021]. Available from: https://www.eurosurveillance.org/content/spotlight-influenza-2021

[2] Larrauri A Prosenc Trilar K. Preparing for an influenza season 2021/22 with a likely co-circulation of influenza virus and SARS-CoV-2. Euro Surveill. 2021;26(41):2100975. 10.2807/1560-7917.ES.2021.26.41.2100975 34651574 PMC8518305

[3] World Health Organization Regional Office for Europe (WHO/Europe). Influenza vaccination coverage and effectiveness. Copenhagen: WHO/Europe. [Accessed: Oct 2021]. Available from: https://www.euro.who.int/en/health-topics/communicable-diseases/influenza/vaccination/influenza-vaccination-coverage-and-effectiveness

[4] Council of the European Union. Council Recommendation of 22 December 2009 on seasonal influenza vaccination (2009/1019/EU). Official Journal of the European Union. 2009. L 348/71. [Accessed: Dec 2021]. Available from: http://eur-lex.europa. eu/LexUriServ/LexUriServ.do?uri=OJ:L:2009:348:0071:0072:E N:PDF

[5] Kwon Y Choe YJ Yun JW Kim HK Kim S Chun C Impact of Media Coverage on Influenza Vaccine Coverage in Elderly Individuals from 2020 to 2021 in the Republic of Korea. Vaccines (Basel). 2021;9(4):367. 10.3390/vaccines9040367 33920117 PMC8070596

[6] Australian Government. Department of Health. Influenza (flu) immunization data. Canberra: Australian Government. Department of Health. [Accessed: Oct 2021]. Available from: https://www.health.gov.au/resources/publications/influenza-flu-immunisation-data

[7] Burki TK. Circulation of influenza, RSV, and SARS-CoV-2: an uncertain season ahead. Lancet Respir Med. 2021;9(10):e103. 10.1016/S2213-2600(21)00364-7 34370976 PMC8346240

[8] Baker RE Park SW Yang W Vecchi GA Metcalf CJE Grenfell BT. The impact of COVID-19 nonpharmaceutical interventions on the future dynamics of endemic infections. Proc Natl Acad Sci USA. 2020;117(48):30547-53. 10.1073/pnas.2013182117 33168723 PMC7720203

[9] European Centre for Disease Prevention and Control (ECDC). Seasonal influenza vaccination in EU/EEA Member States. Stockholm: ECDC. [Accessed: Nov 2021]. Available from: https://www.ecdc.europa.eu/sites/default/files/documents/seasonal-influenza-antiviral-use-2018.pdf

